# Metabolic engineering of *Ustilago trichophora* TZ1 for improved malic acid production

**DOI:** 10.1016/j.meteno.2017.01.002

**Published:** 2017-01-17

**Authors:** Thiemo Zambanini, Hamed Hosseinpour Tehrani, Elena Geiser, Christiane K. Sonntag, Joerg M. Buescher, Guido Meurer, Nick Wierckx, Lars M. Blank

**Affiliations:** a iAMB – Institute of Applied Microbiology, ABBt – Aachen Biology and Biotechnology, RWTH Aachen University, Worringerweg 1, D-52074 Aachen, Germany; b BRAIN AG, Darmstädter Straße 34-36, D-64673 Zwingenberg, Germany; c MPI Immunobiology and Epigenetics, Stübeweg 51, D-79108 Freiburg, Germany

**Keywords:** Carbon-balance, Glycerol, Malate, Metabolic engineering, Overexpression, *Ustilago trichophora*

## Abstract

*Ustilago trichophora* RK089 has been found recently as a good natural malic acid producer from glycerol. This strain has previously undergone adaptive laboratory evolution for enhanced substrate uptake rate resulting in the strain *U. trichophora* TZ1. Medium optimization and investigation of process parameters enabled titers and rates that are able to compete with those of organisms overexpressing major parts of the underlying metabolic pathways. Metabolic engineering can likely further increase the efficiency of malate production by this organism, provided that basic genetic tools and methods can be established for this rarely used and relatively obscure species.

Here we investigate and adapt existing molecular tools from *U. maydis* for use in *U. trichophora*. Selection markers from *U. maydis* that confer carboxin, hygromycin, nourseothricin, and phleomycin resistance are applicable in *U. trichophora*. A plasmid was constructed containing the *ip*-locus of *U. trichophora* RK089, resulting in site-specific integration into the genome. Using this plasmid, overexpression of pyruvate carboxylase, two malate dehydrogenases (*mdh1*, *mdh2*), and two malate transporters (*ssu1*, *ssu2*) was possible in *U. trichophora* TZ1 under control of the strong P_*etef*_ promoter. Overexpression of *mdh1*, *mdh2*, *ssu1*, and *ssu2* increased the product (malate) to substrate (glycerol) yield by up to 54% in shake flasks reaching a titer of up to 120 g L^−1^. In bioreactor cultivations of *U. trichophora* TZ1 P_*etef*_*ssu2* and *U. trichophora* TZ1 P_*etef*_*mdh2* a drastically lowered biomass formation and glycerol uptake rate resulted in 29% (Ssu1) and 38% (Mdh2) higher specific production rates and 38% (Ssu1) and 46% (Mdh2) increased yields compared to the reference strain *U. trichophora* TZ1. Investigation of the product spectrum resulted in an 87% closed carbon balance with 134 g L^−1^ malate and biomass (73 g L^−1^), succinate (20 g L^−1^), CO_2_ (^1^7 g L^−1^), and α-ketoglutarate (8 g L^−1^) as main by-products.

These results open up a wide range of possibilities for further optimization, especially combinatorial metabolic engineering to increase the flux from pyruvate to malic acid and to reduce by-product formation.

## Highlights

• Metabolic engineering tools established for *U. trichophora*
• Potentially carbon neutral process through CO_2_ co-metabolism
• Optimized malic acid production from glycerol by overexpression of rTCA cycle
• Specific production rate and yield increased up to 38% and 46%, respectively

## 1 Introduction

The biotechnological production of chemicals has gained great interest in the last decades. Strongly fluctuating oil prices, environmental pollution, and climate change, have driven the development of new sustainable microbial production processes (Goldberg et al., 2006). One promising group of chemicals are organic acids such as succinic, fumaric, citric, itaconic, and malic acid. As natural metabolites produced by many organisms, the production of these chemicals with a broad range of microbes has been investigated, including different *Candida* species (West, 2013), *Yarrowia lipolytica* (Liu et al., 2015), and *Aspergillus niger* (Xu et al., 1989) for citric acid, *A. terreus* (Klement and Büchs, 2013, Okabe et al., 2009, Steiger et al., 2013) and different *Ustilago* species (Geiser et al., 2016, Geiser et al., 2014, Guevarra and Tabuchi, 1990a, Guevarra and Tabuchi, 1990b, Klement et al., 2012) for itaconic acid, *Rhizopus oryzae* (Rhodes et al., 1962) and *Torulopsis glabrata* (Chen et al., 2015, Chen et al., 2013) for fumaric acid, *Y. lipolytica* (Yuzbashev et al., 2011), *Lactobacillus* species (Kaneuchi et al., 1988) and *Actinobacillus succinogenes* (Guettler et al., 1999, Song and Lee, 2006) for succinic acid, and *Aspergillus* species (Knuf et al., 2013, West, 2011) and *Saccharomyces cerevisiae* (Zelle et al., 2008) for malic acid. Many of these organisms underwent considerable metabolic engineering in order to establish efficient production of the desired chemical.

Metabolic engineering has great potential to improve microbial production processes (Choi et al., 2015). For malic acid production this has been demonstrated in different organisms. In *S. cerevisiae* combined overexpression of the native pyruvate carboxylase gene *pyc2*, an allele of the peroxisomal malate dehydrogenase gene *mdh3*, which had been retargeted to the cytosol by deletion of the C-terminal targeting sequence, and expression of the *Schizosaccharomyces pombe* malate transporter gene *mae1* resulted in a malic acid titer of 59 g L^−1^ produced with a yield of 0.42 mol_mal_ mol_glu_^−1^ (Zelle et al., 2008). By overexpression of a native C4-dicarboxylate transporter, Brown *et al*. were able to improve the malic acid production rate of *A. oryzae* by more than two-fold (Brown et al., 2013). Combined overexpression of cytosolic pyruvate carboxylase and malate dehydrogenase increased the rate by an additional 27%. The final strain overexpressing all conversion and transport steps from pyruvate to extracellular malic acid reached a 2.6-fold increased titer of 154 g L^−1^ produced at a rate of 0.94 g L^−1^ h^−1^ reaching a yield of 1.38 mol mol^−1^ on glucose (Brown et al., 2013). This clearly demonstrates the importance of the reductive tricarboxylic acid (rTCA) pathway among the five identified possible microbial production pathways for malic acid (Zelle et al., 2008). This pathway starts with carboxylation of pyruvate to oxaloacetate by pyruvate carboxylase, which is followed by reduction to malate by malate dehydrogenase (Brown et al., 2013, Zelle et al., 2008). Also in other organisms, such as different *Aspergillus* species and *R. oryzae*, this pathway has been shown to be essential in efficient malic acid production (Bercovitz et al., 1990, Goldberg et al., 2006, Osmani and Scrutton, 1983, Peleg et al., 1988).

As can be seen from the examples above, the majority of microbial production processes still focusses of glucose as substrate. However, different other substrates have gained increasing interest over the last decades. Recently, glycerol, as main low-value by-product of biodiesel production (10% (w/v)), has been proposed as potential substrate for biotechnological production processes (West, 2012, Yang et al., 2012). The still increasing biodiesel production (123 million tons predicted for 2016) and the resulting glycerol side stream, has resulted in intensive search of novel biocatalysts. So far, different production processes have been reported, such as the production of lipids (Saenge et al., 2011), polyols (Rymowicz et al., 2009), and organic acids (Papanikolaou et al., 2003, Scholten et al., 2009). In 2016, we reported *U. trichophora* RK089 as promising production organism for malic acid from glycerol. Adaptive laboratory evolution resulted in strain *U. trichophora* TZ1 with a 6.6-fold increased production rate. After medium optimization a titer of nearly 200 g L^−1^ was reached produced at a rate of 0.39 g L^−1^ h^−1^ (Zambanini et al., 2016c). In bioreactors the production rate was further improved to a maximum of nearly 2 g L^−1^ h^−1^. However, the reached yield was only 31% of the theoretical maximum (Zambanini et al., 2016b), indicating considerable room for improvement. In this study we combine the already high production capability of this genetically unmodified strain with the possible positive effects of overexpressing rTCA pathway genes. However, molecular tools and methods, such as vectors, promoters and terminators for overexpression, applicable antibiotics with corresponding resistance cassettes, and transformation and screening protocols, were not available for this relatively obscure organism. Yet, these tools are known for the model Ustilaginacea *U. maydis* (Geiser et al., 2013, Khrunyk et al., 2010, Schuster et al., 2016, Terfrüchte et al., 2014). Additionally, the genome of *U. trichophora* RK089 has recently been sequenced (Zambanini et al., 2016a), providing a key resource for genetic and metabolic engineering.

Here we report on the investigation, adaptation, and development of molecular tools and methods for *U. trichophora* and the use of these to overexpress native genes for a pyruvate carboxylase, two malate dehydrogenases (mitochondrial and cytoplasmic) and two malate transporters. With these modifications, we aimed to increase the flux from glycerol towards malate, to ultimately improve the product on substrate yield. The resulting strains were analyzed in shake flasks and the data were validated in bioreactors.

## 2 Materials and methods

### 2.1 Strains and culture conditions

All strains used and generated in this study are listed in Table 1.

**Table 1:** *U. trichophora* strains used in this study.

**Strain name**	**Description**	**Reference**
RK089	Wildtype strain	(Kellner, 2011)
TZ1	RK089 adapted to glycerol by adaptive laboratory evolution	(Zambanini et al., 2016c)
RK089 pSMUT	RK089 with genomic integration of pSMUT; hygromycin resistant	This study
RK089 pNEBUC	RK089 episomally expressing pNEBUC; carboxin resistant	This study
RK089 pNEBUN	RK089 episomally expressing pNEBUN; nourseothricin resistant	This study
RK089 pNEBUP	RK089 episomally expressing pNEBUP; phleomycin resistant	This study
TZ1 pUTr01	TZ1 with genomic integration of pUTr01; carboxin resistant	This study
TZ1 P_*etef*_*mdh1*	TZ1 with genomic integration of pUTr01-Mdh1; carboxin resistant	This study
TZ1 P_*etef*_*mdh2* (m)	TZ1 with genomic integration of pUTr01-Mdh2 (m); carboxin resistant	This study
TZ1 P_*etef*_*mdh2* (c)	TZ1 with genomic integration of pUTr01-Mdh2 (c); carboxin resistant	This study
TZ1 P_*etef*_*pyc*	TZ1 with genomic integration of pUTr01-Pyc; carboxin resistant	This study
TZ1 P_*etef*_*ssu1*	TZ1 with genomic integration of pUTr01-Ssu1; carboxin resistant	This study
TZ1 P_*etef*_*ssu2*	TZ1 with genomic integration of pUTr01-Ssu2; carboxin resistant	This study

As standard medium, MTM was used as described previously (Zambanini et al., 2016c). As buffer, either 100 mM 2-(N-morpholino)ethanesulfonic acid (MES) or 100 g L^−1^ CaCO_3_ was used. Pharma grade glycerol was used for all experiments.

For drop tests, 5 µL of a YEP-grown overnight culture diluted to a starting OD_600_ of 1 were pipetted onto a YEP-plate containing different concentrations of carboxin, hygromycin, nourseothricin or phleomycin in different dilutions (1, 10^−1^, 10^−2^, 10^−3^) and incubated (7 days, 30 °C).

Screening experiments were performed in 24 deep well plates (Enzyscreen, System Duetz^®^) with 1.5 mL MTM containing 100*g* L^−1^ CaCO_3_ and 0.8*g* L^−1^ glycerol incubated at 30 °C (relative air humidity =80%) shaking at 300 rpm (shaking diameter =50 mm).

Shake flask production experiments (10% filling volume) were conducted in MTM containing 200*g* L^−1^ glycerol and 0.8*g* L^−1^ NH_4_Cl shaking at 200 rpm as described previously (Zambanini et al., 2016c).

Controlled batch cultivations were performed as described previously (Zambanini et al., 2016b). The pH was set to 6.5 and controlled automatically by 10 M NaOH. As medium, MTM containing 200 g L^−1^ glycerol and either 3.2 g L^−1^ NH_4_Cl or 6.4 g L^−1^ with doubled concentration of all other medium components was used.

### 2.2 Analytical methods

All shaken cultures were performed in triplicates. Bioreactor cultivations were performed in duplicates. Shown is the arithmetic mean of the replicates. Error bars and±values indicate deviation from the mean.

OD_600_ determination and HPLC analysis were performed as described previously (Zambanini et al., 2016c).

Fluorescence was measured in black FLUOTRACK 96-well microtiter plates (Greiner Bio-One GmbH, Frickenhausen, Germany) with a Synergy MxF Fluorescence Microplate Reader (BioTek Instruments Inc., Winooski, USA). An excitation wavelength of 485 nm and an emission wavelength of 530 nm were used and the gain was set to 80.

Fluorescence microscopy was performed on a Leica DM6000 B fluorescence microscope (Wetzlar, Germany) using the fluo green filter at a magnification of 630 with an oil-immersion object. An excitation wavelength of 499 nm and emission wavelength of 520 nm were used. Exposure time was set to 200 ms, gain to 10, and intensity to 4.3.

Extracellular lipids, such as mannosylerythritol lipid or ustilagic acid were analyzed by thin-layer chromatography as described previously (Geiser et al., 2014).

### 2.3 Cloning procedures

Standard cloning-related techniques were performed according to Sambrook *et al*. (Sambrook and Russell, 2001). The genome sequence of *U. trichophora* RK089 (accession number: LVYE01000000) was used as reference (Zambanini et al., 2016a). Genomic DNA from *U. trichophora* was isolated as described previously (Hoffman and Winston, 1987). All vectors used and generated in this study are listed in Table 2.

**Table 2:** Vectors used in this study with description of contained elements.

**Plasmid name**	**Description**	**Reference**
pSMUT	Ori ColE1; ampR; Psc; *hph*	(Bölker et al., 1995)
pNEBUC	*ip*^R^-locus; ori ColE1; UARS; ampR	(Weinzierl, 2001)
pNEBUN	natR; ori ColE1; UARS; ampR	(Weinzierl, 2001)
pNEBUP	bleR; ori ColE1; UARS; ampR	(Weinzierl, 2001)
pUMa43	P_*etef*_; *gfp*; T_*nos*_; ori ColE1; ampR; *U. maydis ip*^*R*^-locus	(König, 2008)
pUTr01	pUMa43 with the *U. maydis ip*^*R*^-locus exchanged for the *ip*^*R*^-locus from *U. trichophora* RK089; P_*etef*_*gfp*	This study
pUTr01-Mdh1	pUTr01 with *gfp* exchanged for Mdh1; P_*etef*_*mdh1*	This study
pUTr01-Mdh2 (m)	pUTr01 with *gfp* exchanged for Mdh2 (m); P_*etef*_*mdh2 (m)*	This study
pUTr01-Mdh2 (c)	pUTr01 with *gfp* exchanged for Mdh2 (c); P_*etef*_*mdh2* (c)	This study
pUTr01-Pyc	pUTr01 with *gfp* exchanged for Pyc; P_*etef*_*pyc*	This study
pUTr01-Ssu1	pUTr01 with *gfp* exchanged for Ssu1; P_*etef*_*ssu1*	This study
pUTr01-Ssu2	pUTr01 with *gfp* exchanged for Ssu2; P_*etef*_*ssu2*	This study

For overexpression all genes were cloned into pUTr01 by exchanging *gfp*. For this, the backbone pUTr01 was amplified via PCR using the primer pair pUMa otef-cbx-fw/pUMa otef-cbx-rv. The resulting fragment was digested using *Mlu*I and *Dpn*I. All inserts were amplified via PCR using the primer pairs listed in Table 3.

**Table 3:** Primers used within this study with corresponding sequences and description.

**Primer**	**Sequence/description**
pUMa43-dCBX-fwd	TTGGCGCGCCAATTAGGCCGGCCTTACCCATTATTGAAGC
	Amplification of *ip*-locus from *U. trichophora*

pUMa43-dCBX-rev	CCTTGGCGCGCCAACCTTAATTAAGGTTGAAAAAGGAAGAG
	Amplification of *ip*-locus from *U. trichophora*

pUMa otef-cbx-fw	CGACGCGTCGATTTGCGGCCGCTTTACCGGCTGCAGATCGTTC
	Amplification of the backbone pUMa without *gfp*

pUMa otef-cbx-rv	CGACGCGTCGGACTAGTCGATCGAATTCCTGCAGCC
	Amplification of the backbone pUMa without *gfp*

UT11161+sig_fwd	GCAGGAATTCGATCGACTAGTATGGTCAAGGCTACTGTTATC
	Amplification of *mdh1* from *U. trichophora*

UT11161+sig_rev	TGCAGCCGGTAAAGCGGCCGCTTAAAGGTTGGCAGTGAAC
	Amplification of *mdh1* from *U. trichophora*

UT00403+sig_fwd	GCAGGAATTCGATCGACTAGTATGTTCGCTCGTCAGGCTC
	Amplification of *mdh2* (m) and *mdh2* (c) from *U. trichophora*

UT00403+sig_rev	TGCAGCCGGTAAAGCGGCCGCTCAAGGGTTGGCGGCGAC
	Amplification of *mdh2* (m) from *U. trichophora*

UT00403-sig_fwd	GCAGGAATTCGATCGACTAGTATGGCTTCGGGCGGTATTG
	Amplification of *mdh2* (c) from *U. trichophora*

UT_05271_fwd	CGATCGACTAGTCCGACGCGTATGGGCTTTGGTATCACC
	Amplification of *ssu1* from *U. trichophora*

UT_05271_rev	GCGGCCGCAAATCGACGCGTTTATCTAGAAGGTGAAGCC
	Amplification of *ssu1* from *U. trichophora*

UT_05764_fwd_II	CGATCGACTAGTCCGACGCGTATGTCACCCAACCCCTCG
	Amplification of *ssu2* from *U. trichophora*

UT_05764_rev_II	GCGGCCGCAAATCGACGCGTTTAGGTGAGGGTCGTCATTC
	Amplification of *ssu2* from *U. trichophora*

UT01054_fwd	TGCAGGAATTCGATCCCATGGATGCCCGTCGAGCCCGAG
	Amplification of *pyc* from *U. trichophora*

UT01054_rev	GATCTGCAGCCGGGCGGCCGCTTAGTGCTCAATTTCGCAGAGCAAGTC
	Amplification of *pyc* from *U. trichophora*

fwd-ampII	TCTGACGCTCAGTGGAAC
	Colony-PCR to test for integration into the *U. trichophora* genome

rev-ampII	TGGTGTCGACGTGAATGC
	Colony-PCR to test for integration into the *U. trichophora* genome

The targeting sequences for *mdh1* and *mdh2* were analyzed using a combination of Signal-3 L and TargetP 1.1 (Shen and Chou, 2007).

All plasmids were assembled in *Escherichia coli* and correctness was confirmed by PCR, restriction digest and sequencing via Eurofins Scientific (Ebersberg, Germany).

For transformation of *U. trichophora*, protoplasts were prepared as described previously (Schulz et al., 1990, Tsukuda et al., 1988) or whole cell transformation was performed (Maassen, 2007).

**Fig. 1:**
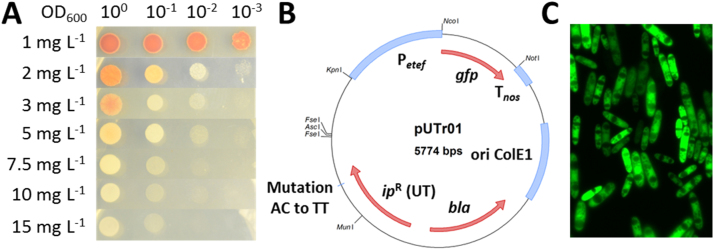
Genetic tool development for *U. trichophora.* A: drop test of 5 µL *U. trichophora* RK089 culture with different dilutions (10°, 10^−1^, 10^−2^, 10^−3^) on YEP plates containing different concentrations of carboxin (1, 2, 3, 5, 7.5, 10, 15 mg L^−1^) B: plasmid map for vector pUTr01. P_*etef*_: *etef* promoter; *gfp*: green fluorescent protein gene; T_*nos*_: *nos*-terminator; ori ColE1: origin of replication in *E. coli*; *bla*: ampicillin resistance cassette; *ip*^R^ (UT): carboxin resistant *ip*-locus of *U. trichophora* C: fluorescence microscopic image of *U. trichophora* RK089 cells expressing pUTr01 after 24 h of cultivation in MTM medium.

The plasmid conferring site-specific integration and resistance to carboxin (cbx) in *U. trichophora,* pUTr01, was constructed by exchanging the cbx-resistant *ip*^R^-locus from *U. maydis* on the plasmid pUMa43 with the *ip*-locus from the genome of *U. trichophora* RK089. For this, the backbone pUMa43 was amplified via PCR with the primer pair pUMa43-dCBX-fwd/pUMa43-dCBX-rev and the resulting fragment was self-circularized after digestion with *Asc*I to give plasmid pUMa43 Δ*ip*^*R*^. The *U. trichophora* specific *ip*-locus was identified based on comparison to the *ip*-locus from *U. maydis* 521. The sequence was point mutated to confer carboxin resistance (position 761–762: AC changed to TT) (Broomfield and Hargreaves, 1992, Keon et al., 1991) and ordered as ‘string’, linear synthetic DNA from Thermo Scientific (Waltham, USA). Additionally, an *Mfe*I/*Mun*I restriction site was added (position 437–438: TG changed to GT). Backbone and insert were assembled using the restriction enzymes *Asc*I and *Pac*I giving plasmid pUTr01 (Fig. 1B).

## 3 Results and discussion

### 3.1 Establishing tools and methods for genetic engineering of *U. trichophora*

Production rate and titer for the recently discovered natural malic acid producer *U. trichophora* RK089 have been improved drastically by adaptive laboratory evolution, medium optimization and process investigation, while the product yield was still low with only 31% of the theoretical maximum (Zambanini et al., 2016b, Zambanini et al., 2016c). In order to increase the yield of the resulting *U. trichophora* TZ1 by metabolic engineering, existing tools from the closely related *U. maydis* had to be investigated and adapted. Since antibiotics and the corresponding resistance cassettes are the basis of classic metabolic engineering, we performed a drop test on YEP plates containing different concentrations of carboxin (cbx), hygromycin (hyg), nourseothricin (nat), and phleomycin (phl).

Typical concentrations of these antibiotics applied to *U. maydis* are 1–4 mg L^−1^ for cbx (Keon et al., 1991, Mahlert et al., 2006, Przybilla, 2014), 200–400 mg L^−1^ for hyg (Brachmann et al., 2004, Keon et al., 1991, Mahlert et al., 2006, Przybilla, 2014, Tsukuda et al., 1988), 50–300 mg L^−1^ for nat (Gold et al., 1994, Mahlert et al., 2006, Przybilla, 2014), and 50 mg L^−1^ for phl (Gold et al., 1994). We tested concentrations in the range of 1–15 (cbx), 100–500 (hyg), 1–300 (nat), and 1–150 mg L^−1^ (phl). Plates were assessed for growth every 24 h by visual inspection. The results for carboxin after 48 h of incubation are shown exemplarily in Fig. 1A. For *U. trichophora* no growth was observed after 48 h exceeding concentrations of 10 (cbx), 300 (hyg), 100 (nat), and 100 mg L^−1^ (phl). In contrast to *U. maydis*, prolonged incubation (>72 h) resulted in growth of *U. trichophora* even at the highest tested concentrations for cbx, hyg, and phl. Only for nat no growth could be observed at concentrations exceeding 200 mg L^−1^. Thus, after transformation, colonies should be picked after approximately 48 h of cultivation. To test whether transformation of *U. trichophora* is possible and the corresponding selection markers are functional, protoplasts were transformed with the episomally replicating plasmids pNEBUC (cbx resistance cassette), pNEBUN (nat resistance cassette), pNEBUP (phl resistance cassette), and genome-integrated pSMUT (hyg resistance cassette). Resulting colonies on selective medium plates with the respective antibiotics were screened for different incubation times. Plasmid-isolation from protoplasts and re-transformation into *E. coli* followed by re-isolation resulted in the correct plasmid for pNEBUN, pNEBUP, and pSMUT. It should be noted, that for all three plasmid transformations the number of background colonies increased after 72 h of incubation, as it has already been observed earlier for *U. maydis* with nat (Gold et al., 1994). This also correlates with the observations from the drop test. In contrast to the other plasmids, for pNEBUC the background of colonies without the plasmid was high resulting in laborious screening for cbx concentrations below 5 mg L^−1^. For concentrations above 5 mg L^−1^, however, no colonies could be observed. Thus, instead of protoplasts transformation, whole cell transformation was performed, using 5, 10, and 15 mg L^−1^ cbx for selection. This transformation resulted in positive colonies for all concentrations. The discrepancy between transformation via protoplasts and whole cells is likely to result from sensitivity of protoplasts, which has been described previously for different organisms. Some antibiotics act as effective growth inhibitors on yeast protoplasts already in low concentrations (Shockman and Lampen, 1962). Also for *Corynebacterium glutamicum* it was shown that an increasing concentration of penicillin reduces the regeneration frequency of protoplasts after transformation (Katsumata et al., 1984).

The tested pNEBUC, pNEBUN, and pNEBUP are self-replicating plasmids. For industrial application, however, a plasmid that integrates into the genome is preferable, since no addition of antibiotics into the medium is needed for plasmid maintenance. The plasmid pSMUT randomly integrates into the genome. Yet, with site-specific integration, screening efforts can be reduced, since unspecific integration likely results in random disruption of unknown genes, or site-specific variation of the expression level. In *U. maydis*, the plasmid pUMa43 confers resistance to carboxin by site-specific integration into the *ip*^S^-locus (König, 2008). The transformation of this plasmid into *U. trichophora* RK089 resulted in positive clones containing the plasmid. However, this construct was not integrated site-specifically.

Since the integration method relies on homologous recombination, it is likely that the 88% DNA sequence homology between *U. maydis’* (sequence donor) and *U. trichophora's* (sequence acceptor) *ip*-locus is too low to ensure site-specific integration. Thus, the *ip*^R^-locus from *U. maydis* on the plasmid pUMa43 was exchanged with the *ip*^R^-locus from *U. trichophora* RK089 resulting in plasmid pUTr01 (Fig. 1B). Site-specificity was confirmed by Southern Blot and PCR. Since this vector harbors *gfp* under control of P_*etef*_, which is known to promote overexpression in *U. maydis* (Sarkari et al., 2014, Spellig et al., 1996), we monitored fluorescence of transformants in microtiter-plates using a microtiter-plate-reader and with fluorescence microscopy. All investigated transformants showed strong fluorescence (Fig. 1C), while the reference strain without plasmid did not, confirming the activity of the expression cassette with P_*etef*_ and T_*nos*_. Thus, the function of all relevant elements of pUTr01 was confirmed. This plasmid enables overexpression of target genes through site-specific genomic integration in *U. trichophora*.

### 3.2 Overexpression of *mdh* and *ssu* increases yield in shake flasks

**Fig. 2:**
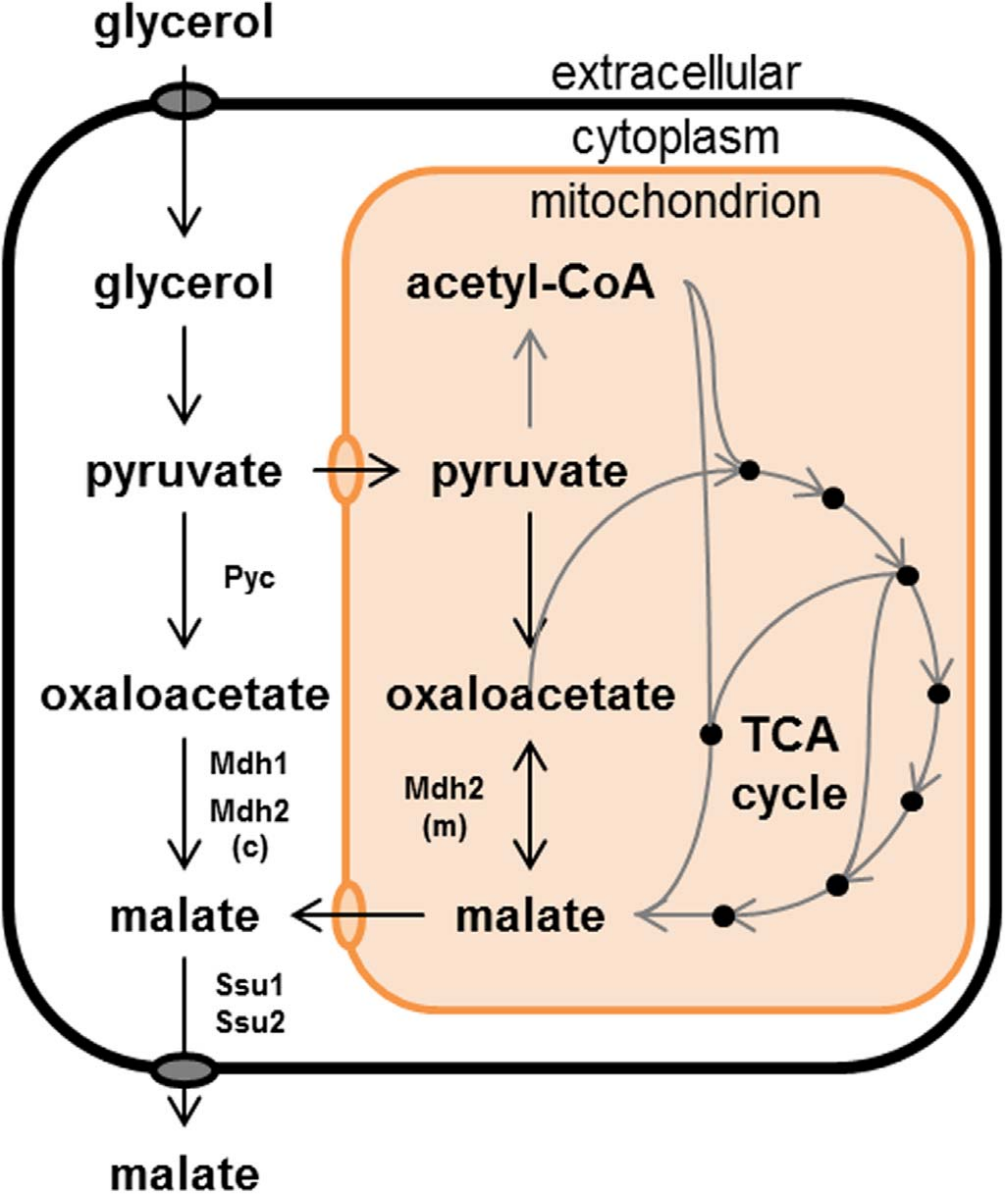
Overexpression targets for metabolic engineering of the reductive TCA cycle for enhanced malate production. Pyc: pyruvate carboxylase; Mdh: malate dehydrogenase; Ssu: malate transporter, (c): cytosolic, and (m): mitochondrial.

With the established tools and methods, optimization of malic acid production by overexpression of expected bottleneck genes in *U. trichophora* TZ1 became possible. As targets we chose all putative enzymes in the reductive tricarboxylic acid (rTCA) cycle leading from pyruvate to malic acid (Fig. 2). Thus, we compared using the Blast-analysis tools (Altschul et al., 1990) the sequences of pyruvate carboxylase UMAG_01054 (Pyc), the two malate dehydrogenases UMAG_11161 (Mdh1) and UMAG_00403 (Mdh2), and the two enzymes related to malic acid transport proteins UMAG_05271 (Ssu1) and UMAG_05764 (Ssu2) from *U. maydis* against the recently published genome of *U. trichophora* (Zambanini et al., 2016a). The search on protein level yielded one hit each for Pyc (97% homology), Mdh1 (97% homology), Mdh2 (94% homology), Ssu1 (89% homology), and Ssu2 (68% homology). For the malate dehydrogenases N-terminal targeting sequences were analyzed using a combination of Signal-3L and TargetP 1.1 (Shen and Chou, 2007). The putative localization for Mdh1 was the cytosol and for Mdh2 the mitochondrion. The N-terminal mitochondrial targeting sequence for *mdh2* was either retained or removed, resulting in the gene versions *mdh2* (m) and *mdh2* (c), which are likely targeted to the mitochondria (m) and the cytosol (c), respectively. All genes were cloned under control of P_*etef*_ into vector pUTr01, by replacing *gfp*. The resulting constructs were transformed into *U. trichophora* TZ1.

A first screening in MTM containing 200 g L^−1^ glycerol in 24-deep well plates revealed a broad variety among resulting mutants after 384 h of cultivation concerning growth, malic acid production, and glycerol uptake (data not shown). From this screening the two best transformants for each gene were chosen for more detailed shake flask investigation. Genomic integrations of the constructs were verified by PCR using the primer pair fwd-ampII/rev-ampII.

Both transformants overexpressing pyruvate carboxylase and both strains overexpressing malate dehydrogenase *mdh2* (c) showed lower or similar malic acid production compared to the reference strain (see supplemental data). This hints at naturally strong activity of *pyc* in *U. trichophora* TZ1 and a bottleneck in another step of the production pathway. Further, the fact that malic acid production did not improve upon overexpression of *mdh2* (c) likely indicates that the gene product of this shorter version of the gene either lacks activity or is no longer localized in the compartment, where it benefits malate production. In general, microbial malic acid production is possible via five different pathways: (1) cytosolic rTCA cycle, (2) mitochondrial rTCA cycle (3) TCA cycle, (4) glyoxylate route (cyclic), (5) glyoxylate route (non-cyclic), which have been discussed in literature (Brown et al., 2013, Zelle et al., 2008).

**Fig. 3:**
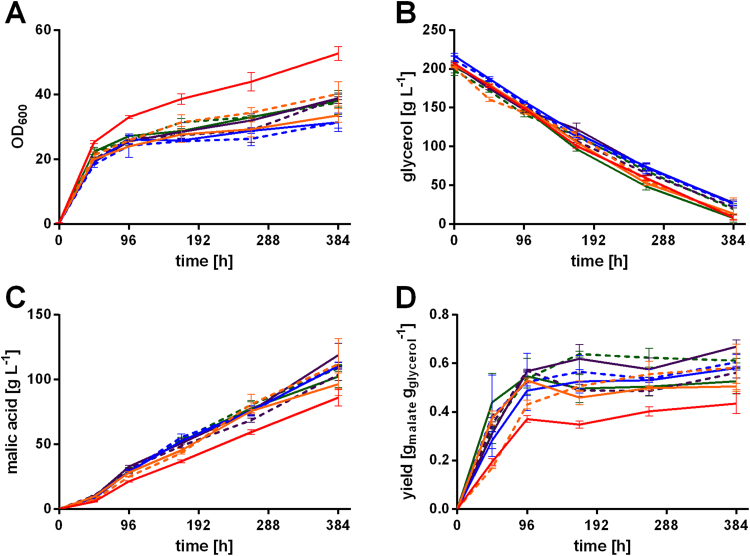
Shake flask cultivation of *U. trichophora* TZ1 mutants in MTM with 200 g L^−1^ glycerol. Cultures contained 100 g L^−1^ CaCO_3_. A: OD_600_, B: glycerol concentration, C: malic acid concentration, and D: cumulative yield over time for *U. trichophora* TZ1 (red) and mutants *U. trichophora* TZ1 P_*etef*_*ssu1* (orange), P_*etef*_*ssu2* (blue), P_*etef*_*mdh1* (purple), and P_*etef*_*mdh2* (m) (green). For each gene two individual transformants (dashed, solid) were investigated. Error bars indicate deviation from the mean of biological replicates of each transformant (n=3). (For interpretation of the references to color in this figure legend, the reader is referred to the web version of this article.)

These pathways do not only differ in the enzymes involved, but also in their subcellular localization. However, the cytosolic rTCA-cycle, comprising the reaction of pyruvate to malic acid via oxaloacetate catalyzed by pyruvate carboxylase and malate dehydrogenase has been reported to be the predominant pathway for extracellular malic acid accumulation in many different organisms (Bercovitz et al., 1990, Brown et al., 2013, Goldberg et al., 2006, Osmani and Scrutton, 1983, Peleg et al., 1988, Zelle et al., 2008), likely also being predominant in *U. trichophora*. Yet, this does not exclude the possibility of an activity of the mitochondrial alleles of malate dehydrogenase, especially since not only overexpression of the gene encoding the cytoplasmic isoenzyme Mdh1 but also of the gene encoding the mitochondrial one, Mdh2 (m), resulted in an increased malic acid yield in *U. trichophora* TZ1 (Fig. 3D). Additionally, the obtained yields are relatively low compared to the theoretical yield possible with exclusive activity of rTCA (1.46 g malate per gram glycerol), which further contributes to the assumption of activity in other pathways. The theoretical yields for the other pathways are lower with 0.73 g malate per gram glycerol for the TCA cycle and cyclic glyoxylate route and 0.95 g malate per gram glycerol for the non-cyclic glyoxylate route.

Besides overexpression of the genes encoding malate dehydrogenases Mdh1 and Mdh2 (m), overexpression of the genes encoding the two transporters Ssu1 and Ssu2 resulted in a 16–54% increased malic acid yield at the end of cultivation (Fig. 3D, supplemental data), reaching a maximal overall yield of 0.67 g_mal_ g_gly_^−1^ for one mutant overexpressing *mdh1* (Fig. 3D, supplemental data). Additionally to an increased yield, optical density in all mutants was lower and the malic acid titer was higher than for the reference strain (Fig. 3A, C), resulting in drastically improved specific malic acid production (g_mal_ OD_600_^−1^). Clearly, the conversion of oxaloacetate to malate by malate dehydrogenase and the export of malic acid seem to be rate limiting steps for *U. trichophora* TZ1. These steps have already been observed in *S. cerevisiae* and *A. oryzae* as being limiting. Single overexpression of malate dehydrogenase and malate permease resulted in a nearly 3-fold increased malic acid production in *S. cerevisiae* (Zelle et al., 2008). For *A. oryzae* overexpression of a C4-dicarboxylic acid transporter resulted in a 2-fold increase, while single overexpression of pyruvate carboxylase did not drastically improve malic acid production (Brown et al., 2013).

### 3.3 Improved yield and specific production rate in bioreactor cultivations with *U. trichophora* TZ1 P_*etef*_*mdh2* (m) and TZ1 P_*etef*_*ssu2*

**Fig. 4:**
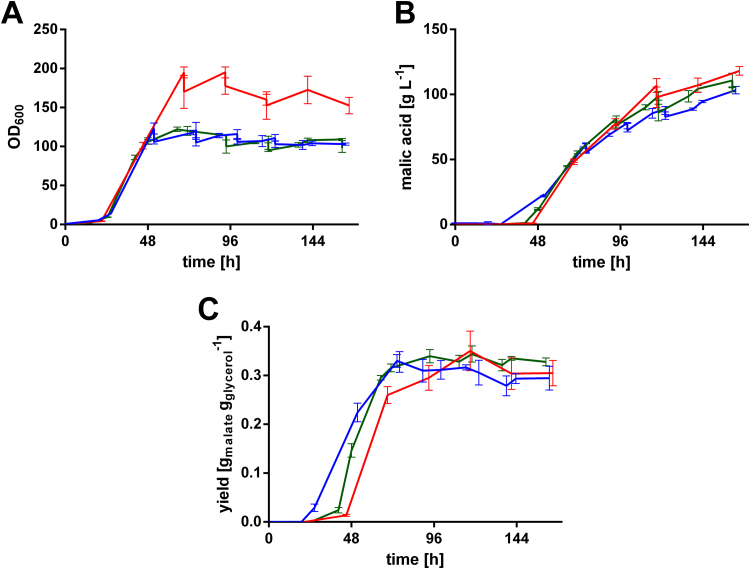
Fermentation of *U. trichophora* TZ1 mutants in doubled MTM. A: OD_600_, B: malic acid concentration, and C: cumulative yield for *U. trichophora* TZ1 (red) (Zambanini et al., 2016b), *U. trichophora* TZ1 P_*etef*_*ssu2* (blue), and *U. trichophora* TZ1 P_*etef*_*mdh2* (m) (green) in controlled batch fermentations in MTM containing 200 g L^−1^ initial glycerol, 6.4 g L^−1^ NH_4_Cl and doubled concentration of all other medium components at 30 °C with DO kept at 80% and pH 6.5 kept constant by automatic NaOH addition. Error bars indicate deviation from the mean (n=2). (For interpretation of the references to color in this figure legend, the reader is referred to the web version of this article.)

Even though, the malic acid titer for Ssu and Mdh transformants was increased compared to *U. trichophora* TZ1, the values are generally lower than previously published titers for *U. trichophora* TZ1 (Zambanini et al., 2016c). These differences result from longer oxygen-limitation during sampling, due to higher sampling efforts with many shake flasks. The strong, negative effect of oxygen limitation on organic acid production has been discussed in literature (Guevarra and Tabuchi, 1990b, Gyamerah, 1995). To overcome these issues (insufficient mixing, oxygen limitation, substrate depletion), and to test whether the observed improvements would hold up under industrially more relevant conditions, *U. trichophora* TZ1 P_*etef*_*ssu2* and TZ1 P_*etef*_*mdh2* (m) were cultivated in bioreactors containing doubled MTM with 200 g L^−1^ initial glycerol and 6.4 g L^−1^ NH_4_Cl (Fig. 4).

**Fig. 5:**
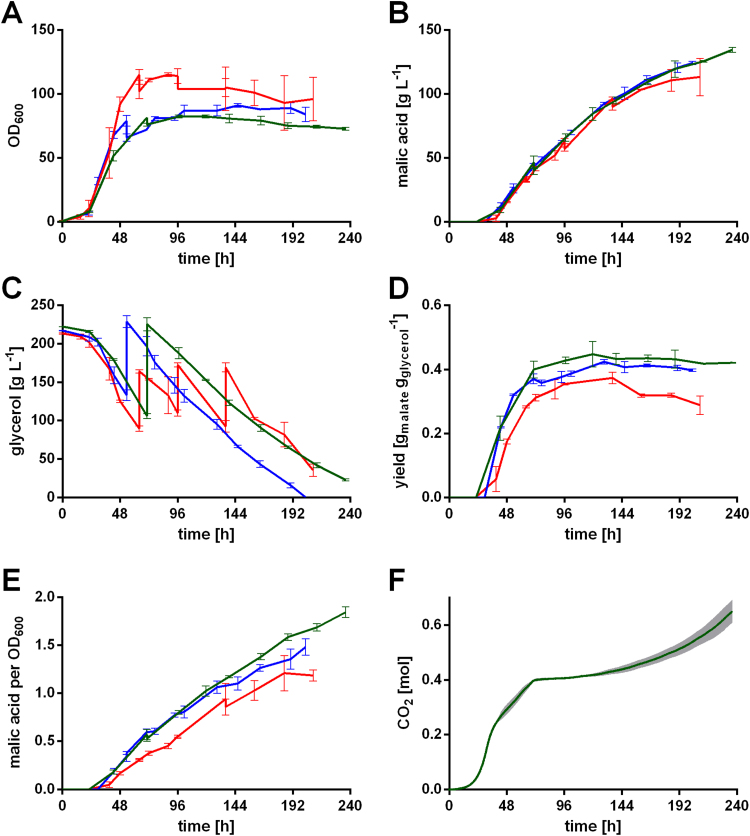
Fermentation of *U. trichophora* TZ1 mutants in MTM. A: OD_600_, B: malic acid concentration, C: glycerol concentration, D: cumulative yiel d, and E: cumulative specific malic acid production for *U. trichophora* TZ1 (red) (Zambanini et al., 2016b), *U. trichophora* TZ1 P_*etef*_*ssu2* (blue), and *U. trichophora* TZ1 P_*etef*_*mdh2* (m) (green) in controlled batch fermentations in MTM containing 200 g L^−1^ initial glycerol, 3.2 g L^−1^ NH_4_Cl at 30 °C with DO kept at 80% and pH 6.5 kept constant by automatic NaOH addition. Sudden changes in glycerol concentration and OD_600_ result from additional glycerol feeds. F: Accumulated amount of CO_2_ for *U. trichophora* TZ1 P_*etef*_*mdh2* (m). Error bars (grey area in F) indicate deviation from the mean (n=2). (For interpretation of the references to color in this figure legend, the reader is referred to the web version of this article.)

The average malic acid production rates in bioreactors (*mdh2* (m): 0.69±0.03 g L^−1^ h^−1^; *ssu2*: 0.63±0.02 g L^−1^ h^−1^) were comparable to the one reached with the reference strain *U. trichophora* TZ1 (0.72±0.02 g L^−1^ h^−1^) (Fig. 4B). Combined with drastically lowered optical densities (Fig. 4A), the specific production rates (g_mal_ OD_600_^−1^ h^−1^) were improved by 1.4-fold. Strikingly, a higher product yield could only be observed until approximately 72 h of cultivation, possibly resulting from an earlier onset of malic acid production (Fig. 4C). The overall product yield for the mutant strains, however, was comparable to the one in the reference strain (Fig. 4B), even though in shake flask cultivations it was significantly increased (Fig. 3D). This observation might be explained by the higher biomass formation, due to an elevated nitrogen concentration compared to shake flasks. Already in previous studies with *U. trichophora* TZ1 in bioreactors, 6.4 g L^−1^ NH_4_Cl had a considerable negative impact on the malic acid yield and a relatively small positive impact on the production rate compared to cultivations containing 3.2 g L^−1^ NH_4_Cl (Zambanini et al., 2016b). In this context we elaborated on the trade-off between yield and production rate resulting from higher biomass concentration. This trade-off is of special importance, since malic acid production only occurs upon nitrogen limitation (Knuf et al., 2013, Peleg et al., 1988), and a high biomass (nitrogen) concentration is needed for elevated production rates. Besides this trade-off, the high concentration of all medium components, combined with the high biomass formation, might trigger stress responses in the cells. This stress could result in a lowered malic acid titer, even though the specific production rate is still increased. To test this hypothesis, the bioreactor cultivation with *U. trichophora* TZ1 P_*etef*_*mdh2* and *U. trichophora* TZ1 P_*etef*_*ssu2* was repeated with MTM containing 3.2 g L^−1^ NH_4_Cl and the normal concentration for all other components (Fig. 5).

As expected with less NH_4_Cl, glycerol uptake in the mutant strains was slower than in the reference strain (Fig. 5C), correlating with our previous observations. Also in this cultivation, optical density was drastically lowered for the mutant strains (Fig. 5A) resulting in a 29% (*ssu2*) and 38% (*mdh2*) increased specific malic acid production (Fig. 5E). Yet, as previously discussed (Klement et al., 2012, Maassen et al., 2013, Zambanini et al., 2016c), a lower optical density for Ustilaginaceae does not necessarily imply a lower concentration of active biomass. However, the simultaneously reduced glycerol uptake strengthens the possibility of actually lowered active biomass. Combined with a slightly increased malic acid titer (Fig. 5B), the overall yield was improved by 1.4-fold (*ssu2*) and 1.5-fold (*mdh2*) to 0.40±0.00 g_mal_ g_gly_^−1^ and 0.42±0.00 g_mal_ g_gly_^−1^, respectively (Fig. 5D).

Even though the yield could be improved, these values represent just about 30% of the theoretical maximum (1.46 g_mal_ g_gly_^−1^). Previously we published a yield of 31% for *U. trichophora* TZ1, which however resulted from bioreactor cultivations with CaCO_3_ as buffering agent (Zambanini et al., 2016b). As discussed in this context, the CaCO_3_ serves as buffering agent, *in-situ* calcium malate precipitation alleviates product inhibition, and most importantly, it supplies additional CO_2_ which is required for the operation of pyruvate carboxylase (Battat et al., 1991, Brown et al., 2013, Zambanini et al., 2016b). In the present bioreactor cultivations, NaOH was used for titration. The resulting limitation of CO_2_ during the production phase can clearly be seen from the accumulated amount of CO_2_ during the production process (Fig. 5F). While the CO_2_ production rate during the growth phase is high, it drops to nearly zero during mid-production phase after biomass formation. In this phase, the CO_2_ concentration in the off-gas is below 0.05%, indicating that a higher malic acid yield could be achieved if additional CO_2_ was supplied. At the end of the acid production phase the release of CO_2_ increases again, possibly due to lower malic acid production resulting from product inhibition. These data indicate that CO_2_ co-metabolism is important for reaching a high yield by efficient operation of the rTCA-cycle. This is further supported by the fact that a switch from NaOH to CaCO_3_ as buffering agent increased the product yield by 1.5-fold in a previous study (Zambanini et al., 2016b). However, this beneficial effect of CaCO_3_ as buffering agent does not solely result from additional CO_2_ supply but is rather a combination of CO_2_ co-feed for an increased malic acid yield and decreased product inhibition due to calcium malate precipitation resulting in a higher malic acid production rate.

Thus, application of CaCO_3_ in bioreactor cultures of *U. trichophora* TZ1 P_*etef*_*ssu2* would likely result in a further improved malic acid yield due to the additionally supplied CO_2_. Further, the production rate and titer would be improved due to decreased product inhibition. Assuming an improvement of 1.5-fold for the malic acid yield as result of switching to CaCO_3_, just as reported for *U. trichophora* TZ1 (Zambanini et al., 2016b), the yield would be improved to about 45% of the theoretical maximum. Compared to the yield achieved for malic acid production from glucose with *A. oryzae* (69% of the theoretical) (Brown et al., 2013), this value still indicates a considerable loss of carbon source.

### 3.4 Biomass, succinate, and CO_2_ as main by-products

In order to identify possible targets for further improvement, we determined all measurable by-products and quantified the amount of contained carbon source for the bioreactor cultivation with *U. trichophora* P_*etef*_*mdh2* (m). HPLC-analysis revealed 19.5±0.4 g L^−1^ succinate, 8.4±0.3 g L^−1^ α-ketoglutarate, 1.6±0.4 g L^−1^ fumarate, 1.1±0.1 g L^−1^ *cis*-aconitate, and 0.1±0.0 g L^−1^ oxalate as by-products. Together with 72.8±0.1 g L^−1^ biomass (based on the composition determined by Klement *et al*. (Klement et al., 2012) and 16.7±1.1 g L^−1^ CO_2_, mass balancing accounted for 87.0±0.3% of the added 319.1±3.9 g L^−1^ glycerol (10.4±0.1 Cmol L^−1^) indicating that 1.4±0.1 Cmol L^−1^ (2.3±0.1 Cmol in total) were still unaccounted for. Since all peaks in HPLC-analysis were identified, the production of further organic acids or polyols is unlikely. The production of extracellular glycolipids, such as mannosylerythritol lipid and ustilagic acid, is known for Ustilaginaceae from literature and may explain the missing carbon fraction (Geiser et al., 2014, Hewald et al., 2006, Klement et al., 2012, Morita et al., 2009, Teichmann et al., 2007). Thin-layer chromatography, however, did not reveal any extracellular lipid formation for *U. trichophora* TZ1 (data not shown). With further organic acids, polyols and extracellular lipids excluded as by-products, intracellular lipids accounted for in the biomass and CO_2_ determined the unaccounted 2.3±0.1 Cmol should be further investigated.

Even though we were not able to close the carbon balance completely, the results show, that the main by-product, biomass, can only be lowered by a simultaneous reduction of the production rate. To overcome the reduced yield resulting from biomass formation, cell recycling or immobilization would be promising strategies. These techniques can increase the uptime of the production process drastically and compensate for the negative effect from strong biomass formation. Several studies already reported on the possibility of organic acid production with immobilized cells (Kautola et al., 1985, Kautola et al., 1990, Takata et al., 1980, Willke and Vorlop, 2001). In a previous study, we demonstrated the extreme stability of *U. trichophora* TZ1 in bioreactors. A culture with one eighth of nitrogen supplied was able to reach the same titer as the reference culture with a constant production rate for more than 900 h (Zambanini et al., 2016b). This clearly demonstrates that the development and investigation of the production process has to go hand in hand with the generation of the production organism. Consequently, further optimization should focus both on the reduction of the other organic acids (succinate, α-ketoglutarate) and development of modern process techniques which benefit from the robustness of *U. trichophora* TZ1, to overcome the drawbacks of high cell density cultivations and establish *U. trichophora* as novel industrial production organism for malic acid.

## 4 Conclusions

The potential of overexpressing rTCA in parts or in total for improved malic acid production has been shown consistently over the last years in different organisms. Also in *U. trichophora* TZ1 the single overexpression of genes from this pathway resulted in increased malic acid production, with simultaneously reduced glycerol uptake for nearly all mutants, thus improving the yield by more than 1.5-fold in shake flasks and 1.4-fold in bioreactors. Interestingly, overexpression of malic acid transporters and of malate dehydrogenases results in drastically lowered biomass formation, increasing the specific production rate by up to 1.4-fold. Further improvements can be expected by combinatorial overexpression of all three genes to further increase the flux of glycerol in favor of malic acid production with *U. trichophora* TZ1. This would further contribute to establish an industrially feasible malic acid production process from glycerol with *U. trichophora* TZ1, which could potentially be carbon negative due to the co-metabolism of CO_2_. With such a process, the efficiency of bio-diesel refineries could be further improved, thus contributing to the overall concept of a sustainable and innovative bioeconomy.

## Competing interests

GM is paid employee of BRAIN AG. JMB was paid employee of BRAIN AG. The authors declare that no financial or non-financial conflict of interest was present with regard to the results or interpretation of the reported experiments. Further, they declare that this does not alter the permission of unrestricted use, distribution, and reproduction in any medium, provided the original author and source are credited.

## Funding

This study was partially funded by Biotechnology Research And Information Network AG (BRAIN AG) and by the German Federal Ministry of Education and Research (BMBF) as part of the Strategic Alliance ZeroCarbFP (grant no. FKZ 031A217F).

## Author's contributions

L.M.B., N.W. and G.M. conceived and designed the project. T.Z., N.W., J.M.B and L.M.B. designed experiments and analyzed results. T.Z. and N.W. wrote the manuscript with the help of L.M.B. and J.M.B. H.T., E.G., C.K.S and T.Z. constructed the strains and performed screening experiments. H.T., E.G. and T.Z. performed bioreactor cultivations. All authors read and approved the manuscript.

## References

[bib1] Altschul S.F., Gish W., Miller W., Myers E.W., Lipman D.J. (1990). Basic local alignment search tool. J. Mol. Biol..

[bib2] Battat E., Peleg Y., Bercovitz A., Rokem J.S., Goldberg I. (1991). Optimization of l-malic acid production by *Aspergillus flavus* in a stirred fermentor. Biotechnol. Bioeng..

[bib3] Bercovitz A., Peleg Y., Battat E., Rokem J.S., Goldberg I. (1990). Localization of pyruvate carboxylase in organic acid-producing *Aspergillus* strains. Appl. Environ. Microbiol..

[bib4] Bölker M., Bohnert H.U., Braun K.H., Gorl J., Kahmann R. (1995). Tagging pathogenicity genes in *Ustilago maydis* by restriction enzyme-mediated integration (REMI). Mol. Gen. Genet..

[bib5] Brachmann A., König J., Julius C., Feldbrügge M. (2004). A reverse genetic approach for generating gene replacement mutants in *Ustilago maydis*. Mol. Genet. Genom..

[bib6] Broomfield P.L., Hargreaves J.A. (1992). A single amino-acid change in the iron-sulphur protein subunit of succinate dehydrogenase confers resistance to carboxin in *Ustilago maydis*. Curr. Genet..

[bib7] Brown S.H., Bashkirova L., Berka R., Chandler T., Doty T., McCall K., McCulloch M., McFarland S., Thompson S., Yaver D., Berry A. (2013). Metabolic engineering of *Aspergillus oryzae* NRRL 3488 for increased production of L-malic acid. Appl. Microbiol. Biotechnol..

[bib8] Chen X., Wu J., Song W., Zhang L., Wang H., Liu L. (2015). Fumaric acid production by *Torulopsis glabrata*: engineering the urea cycle and the purine nucleotide cycle. Biotechnol. Bioeng..

[bib9] Chen X., Xu G., Xu N., Zou W., Zhu P., Liu L., Chen J. (2013). Metabolic engineering of *Torulopsis glabrata* for malate production. Metab. Eng..

[bib10] Choi S., Song C.W., Shin J.H., Lee S.Y. (2015). Biorefineries for the production of top building block chemicals and their derivatives. Metab. Eng..

[bib11] Geiser E., Przybilla S.K., Engel M., Kleineberg W., Buttner L., Sarikaya E., Hartog T.D., Klankermayer J., Leitner W., Bolker M., Blank L.M., Wierckx N. (2016). Genetic and biochemical insights into the itaconate pathway of *Ustilago maydis* enable enhanced production. Metab. Eng..

[bib12] Geiser E., Wiebach V., Wierckx N., Blank L.M. (2014). Prospecting the biodiversity of the fungal family Ustilaginaceae for the production of value-added chemicals. BMC Fungal Biol. Biotechnol..

[bib13] Geiser E., Wierckx N., Zimmermann M., Blank L.M. (2013). Identification of an endo-1,4-beta-xylanase of *Ustilago maydis*. BMC Biotechnol..

[bib14] Gold S.E., Bakkeren G., Davies J.E., Kronstad J.W. (1994). Three selectable markers for transformation of *Ustilago maydis*. Gene.

[bib15] Goldberg I., Rokem J.S., Pines O. (2006). Organic acids: old metabolites, new themes. J. Chem. Technol. Biotechnol..

[bib16] Guettler M.V., Rumler D., Jain M.K. (1999). *Actinobacillus succinogenes* sp. nov., a novel succinic-acid-producing strain from the bovine rumen. Int. J. Syst. Bacteriol..

[bib17] Guevarra E.D., Tabuchi T. (1990). Accumulation of itaconic, 2-hydroxyparaconic, itatartaric, and malic acids by strains of the genus *Ustilago*. Agric. Biol. Chem..

[bib18] Guevarra E.D., Tabuchi T. (1990). Production of 2-hydroxyparaconic and itatartaric acids by *Ustilago cynodontis* and simple recovery process of the acids. Agric. Biol. Chem..

[bib19] Gyamerah M.H. (1995). Oxygen requirement and energy relations of itaconic acid fermentation by *Aspergillus terreus* NRRL 1960. Appl. Microbiol. Biotechnol..

[bib20] Hewald S., Linne U., Scherer M., Marahiel M.A., Kämper J., Bölker M. (2006). Identification of a gene cluster for biosynthesis of mannosylerythritol lipids in the basidiomycetous fungus *Ustilago maydis*. Appl. Environ. Microbiol..

[bib21] Hoffman C.S., Winston F. (1987). A ten-minute DNA preparation from yeast efficiently releases autonomous plasmids for transformation of *Escherichia coli*. Gene.

[bib22] Kaneuchi C., Seki M., Komagata K. (1988). Production of succinic acid from citric acid and related acids by *lactobacillus* strains. Appl. Environ. Microbiol..

[bib23] Katsumata R., Ozaki A., Oka T., Furuya A. (1984). Protoplast transformation of glutamate-producing bacteria with plasmid DNA. J. Bacteriol..

[bib24] Kautola H., Vahvaselkä M., Linko Y.Y., Linko P. (1985). Itaconic acid production by immobilized *Aspergillus terreus* from xylose and glucose. Biotechnol. Lett..

[bib25] Kautola H., Vassilev N., Linko Y.Y. (1990). Continuous itaconic acid production by immobilized biocatalysts. J. Biotechnol..

[bib26] Kellner R. (2011). Der Einfluss sexueller Reproduktion und Virulenz auf die Evolution und Speziation der biotrophen Brandpilzfamilie Ustilaginaceae.

[bib27] Keon J.P., White G.A., Hargreaves J.A. (1991). Isolation, characterization and sequence of a gene conferring resistance to the systemic fungicide carboxin from the maize smut pathogen, *Ustilago maydis*. Curr. Genet..

[bib28] Khrunyk Y., Munch K., Schipper K., Lupas A.N., Kahmann R. (2010). The use of FLP-mediated recombination for the functional analysis of an effector gene family in the biotrophic smut fungus *Ustilago maydis*. New Phytol..

[bib29] Klement T., Büchs J. (2013). Itaconic acid - a biotechnological process in change. Bioresour. Technol..

[bib30] Klement T., Milker S., Jäger G., Grande P.M., de Maria P.D., Büchs J. (2012). Biomass pretreatment affects *Ustilago maydis* in producing itaconic acid. Microb. Cell. Fact..

[bib31] Knuf C., Nookaew I., Brown S.H., McCulloch M., Berry A., Nielsen J. (2013). Investigation of malic acid production in *Aspergillus oryzae* under nitrogen starvation conditions. Appl. Environ. Microbiol..

[bib32] König J. (2008). Die Identifikation von Ziel-Transkripten des RNA bindenden Proteins Rrm4 aus *Ustilago maydis*.

[bib33] Liu X., Lv J., Xu J., Zhang T., Deng Y., He J. (2015). Citric acid production in *Yarrowia lipolytica* SWJ-1b yeast when grown on waste cooking oil. Appl. Biochem. Biotechnol..

[bib34] Maassen N. (2007). Gewinnung von Gärungsmutanten der Hefe *Pichia stipitis* durch zufällige Integrationsmutagenese.

[bib35] Maassen N., Panakova M., Wierckx N., Geiser E., Zimmermann M., Bölker M., Klinner U., Blank L.M. (2013). Influence of carbon and nitrogen concentration on itaconic acid production by the smut fungus *Ustilago maydis*. Eng. Life Sci..

[bib36] Mahlert M., Leveleki L., Hlubek A., Sandrock B., Bölker M. (2006). Rac1 and Cdc42 regulate hyphal growth and cytokinesis in the dimorphic fungus *Ustilago maydis*. Mol. Microbiol..

[bib37] Morita T., Fukuoka T., Imura T., Kitamoto D. (2009). Production of glycolipid biosurfactants by basidiomycetous yeasts. Biotechnol. Appl. Biochem..

[bib38] Okabe M., Lies D., Kanamasa S., Park E.Y. (2009). Biotechnological production of itaconic acid and its biosynthesis in *Aspergillus terreus*. Appl. Microbiol. Biotechnol..

[bib39] Osmani S.A., Scrutton M.C. (1983). The sub-cellular localisation of pyruvate carboxylase and of some other enzymes in *Aspergillus nidulans*. Eur. J. Biochem..

[bib40] Papanikolaou S., Muniglia L., Chevalot I., Aggelis G., Marc I. (2003). Accumulation of a cocoa-butter-like lipid by *Yarrowia lipolytica* cultivated on agro-industrial residues. Curr. Microbiol..

[bib41] Peleg Y., Stieglitz B., Goldberg I. (1988). Malic acid accumulation by *Aspergillus flavus*. Appl. Microbiol. Biotechnol.

[bib42] Przybilla S. (2014). Genetische und biochemische Charakterisierung der Itaconsäure-Biosynthese in *Ustilago maydis*.

[bib43] Rhodes R.A., Lagoda A.A., Misenheimer T.J., Smith M.L., Anderson R.F., Jackson R.W. (1962). Production of fumaric acid in 20-liter fermentors. Appl Microbiol..

[bib44] Rymowicz W., Rywinska A., Marcinkiewicz M. (2009). High-yield production of erythritol from raw glycerol in fed-batch cultures of *Yarrowia lipolytica*. Biotechnol. Lett..

[bib45] Saenge C., Cheirsilp B., Suksaroge T.T., Bourtoom T. (2011). Potential use of oleaginous red yeast *Rhodotorula glutinis* for the bioconversion of crude glycerol from biodiesel plant to lipids and carotenoids. Process Biochem..

[bib46] Sambrook J., Russell D.W. (2001). Molecular Cloning: A Laboratory Manual.

[bib47] Sarkari P., Reindl M., Stock J., Muller O., Kahmann R., Feldbrugge M., Schipper K. (2014). Improved expression of single-chain antibodies in *Ustilago maydis*. J. Biotechnol..

[bib48] Scholten E., Renz T., Thomas J. (2009). Continuous cultivation approach for fermentative succinic acid production from crude glycerol by *Basfia succiniciproducens* DD1. Biotechnol. Lett..

[bib49] Schulz B., Banuett F., Dahl M., Schlesinger R., Schafer W., Martin T., Herskowitz I., Kahmann R. (1990). The b alleles of *U. maydis*, whose combinations program pathogenic development, code for polypeptides containing a homeodomain-related motif. Cell.

[bib50] Schuster M., Schweizer G., Reissmann S., Kahmann R. (2016). Genome editing in *Ustilago maydis* using the CRISPR-Cas system. Fungal Genet. Biol..

[bib51] Shen H.B., Chou K.C. (2007). Signal-3L: a 3-layer approach for predicting signal peptides. Biochem. Biophys. Res. Commun..

[bib52] Shockman G.D., Lampen J.O. (1962). Inhibition by antibiotics of the growth of bacterial and yeast protoplasts. J. Bacteriol..

[bib53] Song H., Lee S.Y. (2006). Production of succinic acid by bacterial fermentation. Enzym. Microb. Technol..

[bib54] Spellig T., Bottin A., Kahmann R. (1996). Green fluorescent protein (GFP) as a new vital marker in the phytopathogenic fungus *Ustilago maydis*. Mol. Gen. Genet..

[bib55] Steiger M.G., Blumhoff M.L., Mattanovich D., Sauer M. (2013). Biochemistry of microbial itaconic acid production. Front Microbiol..

[bib56] Takata I., Yamamoto K., Tosa T., Chibata I. (1980). Immobilization of *Brevibacterium flavum* with carrageenan and its application for continuous production of l-malic acid. Enzym. Microb. Technol..

[bib57] Teichmann B., Linne U., Hewald S., Marahiel M.A., Bölker M. (2007). A biosynthetic gene cluster for a secreted cellobiose lipid with antifungal activity from *Ustilago maydis*. Mol. Microbiol..

[bib58] Terfrüchte M., Joehnk B., Fajardo-Somera R., Braus G.H., Riquelme M., Schipper K., Feldbrügge M. (2014). Establishing a versatile Golden Gate cloning system for genetic engineering in fungi. Fungal Genet. Biol..

[bib59] Tsukuda T., Carleton S., Fotheringham S., Holloman W.K. (1988). Isolation and characterization of an autonomously replicating sequence from *Ustilago maydis*. Mol. Cell. Biol..

[bib60] Weinzierl G. (2001). Isolierung und Charakterisierung von Komponenten der b-vermittelten Regulationskaskade in *Ustilago maydis*.

[bib61] West T.P. (2011). Malic acid production from thin stillage by *Aspergillus species*. Biotechnol. Lett..

[bib62] West T.P. (2012). Crude glycerol: a feedstock for organic acid production by microbial bioconversion. J. Microb. Biochem. Technol..

[bib63] West T.P. (2013). Citric acid production by *Candida* species grown on a soy-based crude glycerol. Prep. Biochem. Biotechnol..

[bib64] Willke T., Vorlop K.D. (2001). Biotechnological production of itaconic acid. Appl. Microbiol. Biotechnol..

[bib65] Xu D.B., Madrid C.P., Rohr M., Kubicek C.P. (1989). The influence of type and concentration of the carbon source on production of citric acid by *Aspergillus niger*. Appl. Microbiol. Biotechnol..

[bib66] Yang F., Hanna M.A., Sun R. (2012). Value-added uses for crude glycerol--a byproduct of biodiesel production. Biotechnol. Biofuels.

[bib67] Yuzbashev T.V., Yuzbasheva E.Y., Laptev I.A., Sobolevskaya T.I., Vybornaya T.V., Larina A.S., Gvilava I.T., Antonova S.V., Sineoky S.P. (2011). Is it possible to produce succinic acid at a low pH?. Bioeng. Bugs.

[bib68] Zambanini T., Buescher J.M., Meurer G., Wierckx N., Blank L.M. (2016). Draft genome Sequence of *Ustilago trichophora* RK089, a promising malic acid producer. Genome Announc..

[bib69] Zambanini T., Kleineberg W., Sarikaya E., Buescher J.M., Meurer G., Wierckx N., Blank L.M. (2016). Enhanced malic acid production from glycerol with high-cell-density *Ustilago trichophora* TZ1 cultivations. Biotechnol. Biofuels.

[bib70] Zambanini T., Sarikaya E., Kleineberg W., Buescher J.M., Meurer G., Wierckx N., Blank L.M. (2016). Efficient malic acid production from glycerol with *Ustilago trichophora* TZ1. Biotechnol. Biofuels.

[bib71] Zelle R.M., de Hulster E., van Winden W.A., de Waard P., Dijkema C., Winkler A.A., Geertman J.M., van Dijken J.P., Pronk J.T., van Maris A.J. (2008). Malic acid production by *Saccharomyces cerevisiae*: engineering of pyruvate carboxylation, oxaloacetate reduction, and malate export. Appl. Environ. Microbiol..

